# Delay in referral to hot foot clinic; a root cause analysis and suggestions for service improvement

**DOI:** 10.1186/1753-6561-9-S1-A42

**Published:** 2015-01-14

**Authors:** KT Canavan, A Martin

**Affiliations:** 1 University of Bristol, Western General Hospital, Bristol, UK; 2 Department of Vascular Surgery, Western General Hospital, Bristol, UK

## Background

Approximately 125 diabetes dependent amputations are carried out in the UK each week, which is anticipated to increase by 17% by 2014. With 80% of amputations being preventable, in 2012, NHS diabetes launched a campaign to reduce these figures by 50% by 2018 [1]. In light of this, a multidisciplinary hot foot clinic was established in Weston Area Health Trust. The clinic aim is that of early identification and treatment of foot ulceration and ensuring patients are receiving adequate community follow up, education and orthotics, where needed. The aim of the current project was to assess the care pathway leading to referral to the clinic and performing a root cause analysis on delay in referral.

## Methods

Questionnaires were completed with 10 patients attending the hot foot clinic in two consecutive weeks in Weston General Hospital. The questionnaire assessed; patient factors, pathway of referral to clinic, footcare and education and patient satisfaction. Results of the study were compared with the most recently published Weston Area Health guidelines.

## Results

All 10 patients had type II diabetes. It took an average of 25 days to identify the foot pathology and a further 45 days to be referred onto clinic. Once referred, the average wait for an appointment was 9 days. 60% of patients could comment on how to personally care for their feet and 50% were known to community podiatric services. None could recall their foot risk score from their previous annual review. A root cause analysis identified four areas contributing to a delay in referral to clinic; patient education, staff education, community foot care services and problems surrounding communication amongst the diabetic multidisciplinary team (MDT).

## Conclusions

Foot complications remain a huge burden on the NHS budget and patient quality of life. Employment of guidelines set out by NICE and NHS diabetes is crucial to achieving the 50% reduction in amputations. However, the current study also proposes the development of a combined diabetic care, handheld patient booklet, to allow empowerment of the patients on their condition and improved communication between the members of the diabetic MDT.
